# Data on the solution and processing time reached when constructing a phylogenetic tree using a quantum-inspired computer

**DOI:** 10.1016/j.dib.2023.108970

**Published:** 2023-02-13

**Authors:** Wataru Onodera, Nobuyuki Hara, Shiho Aoki, Toru Asahi, Naoya Sawamura

**Affiliations:** a Faculty of Science and Engineering, Waseda University, TWIns, 2-2 Wakamatsu, Shinjuku, Tokyo 162-8480, Japan; b Fujitsu Ltd. Kanagawa 211-8588, Japan; c Research Organization for Nano & Life Innovation, Waseda University, Japan; d Green Computing Systems Research Organization, Waseda University, Japan

**Keywords:** Phylogenetic reconstruction, Quantum-inspired computing, Distance-matrix method, Graph cut

## Abstract

Phylogenetic trees provide insight into the evolutionary trajectories of species and molecules. However, because (2n-5)! Phylogenetic trees can be constructed from a dataset containing n sequences, but this method of phylogenetic tree construction is not ideal from the viewpoint of a combinatorial explosion to determine the optimal tree using brute force. Therefore, we developed a method for constructing a phylogenetic tree using a Fujitsu Digital Annealer, a quantum-inspired computer that solves combinatorial optimization problems at a high speed. Specifically, phylogenetic trees are generated by repeating the process of partitioning a set of sequences into two parts (*i.e.*, the graph-cut problem). Here, the optimality of the solution (normalized cut value) obtained by the proposed method was compared with the existing methods using simulated and real data. The simulation dataset contained 32–3200 sequences, and the average branch length according to a normal distribution or the Yule model ranged from 0.125 to 0.750, covering a wide range of sequence diversity. In addition, the statistical information of the dataset is described in terms of two indices: transitivity and average p-distance. As phylogenetic tree construction methods are expected to continue to improve, we believe that this dataset can be used as a reference for comparison and confirmation of the validity of the results. Further interpretation of these analyses is explained in W. Onodera, N. Hara, S. Aoki, T. Asahi, N. Sawamura, Phylogenetic tree reconstruction via graph cut presented using a quantum-inspired computer, Mol. Phylogenet. Evol. 178 (2023) 107636.

**Specifications Table**

**Table:** 

Subject	Biological sciences

Specific subject area	Phylogenetic and Evolution
Type of data	Graph, Figure
How the data were acquired	Sequences were simulated from either 1) generated or 2) empirical phylogenetic tree. Phylogenetic trees were generated using the *rtree* function of ape package or the *sim.bd.taxa* function of TreeSim package. Empirical phylogenetic trees were acquired through the RAxML Grove database. Using the trees, sequences were simulated using the INDELible software. Sequences were then subjected to bipartition (graph cut) using second generation of Fujitsu's Digital Annealer or spectral clustering of scikit-learn. The resultant clusters were used to calculate normalized cut value represented as optimality of a solution.
Data format	Analyzed
Description of data collection	The generated phylogenetic tree contained 32 to 3200 sequences, with the average branch length ranging from 0.125 to 0.750 under a normal distribution or the Yule model. Phylogenetic trees retrieved from the RAxML Grove database contained 20 to 40 sequences, with branch length ranging from 0.25 to 0.75. These trees were processed in the INDELible v1.03 software under the WAG+Γ+I substitution model to simulate sequences consisting of 500 residues.
Data source location	Phylogenetic trees were simulated using the ape package or TreeSim package in R. Empirical phylogenetic tree data were downloaded from RAxML Grove database.
Data accessibility	Repository name: Mendeley Data Data identification number: https://doi.org/10.17632/gjyhp3f8f3.1 Direct URL to data: https://data.mendeley.com/datasets/gjyhp3f8f3/1
Related research article	W. Onodera, N. Hara, S. Aoki, T. Asahi, N. Sawamura, Phylogenetic tree reconstruction via graph cut presented using a quantum-inspired computer, Mol. Phylogenet. Evol. 178 (2023) 107636 [1].

## Value of the Data

• These data contain the result for normalized cut of graph-presented sequences using Fujitsu's quantum-inspired computer, Digital Annealer.
• These data can serve as benchmarks for solution optimality when developing a phylogenetic tree construction method based on graph cuts. The solution optimality was evaluated as the normalized cut value used in the field of graph theory.
• These data contain both simulated (Yule model) and real (RAxML Grove database) phylogenetic trees in the newick format, enabling their direct use in the benchmarking of new phylogenetic tree construction methods.
• These data describe how the sequences were formulated into the Ising model (Quadratic Unconstrained Binary Optimization; QUBO formula) to construct a phylogenetic tree using a quantum annealing machine (quantum-inspired machine, digital annealer). The QUBO formula is also used in other annealing machines, and will be valuable for future research.

## 1 Objective

Using quantum-inspired computer, Fujitsu's Digital Annealer (DA), normalized cut value together with processing time derived by cutting graph-presented sequences were compared with existing graph cut methods. These data support the findings of the previously mentioned article (‘Phylogenetic tree reconstruction via graph cut presented using a quantum-inspired computer’ (W. Onodera, N. Hara, S. Aoki, T. Asahi, N. Sawamura. Mol. Phylogenet. Evol. 178 (2023) 107636)) by comparing each cut performance required to construct a phylogenetic tree.

## 2 Data Description

The normalized minimum cut using the Digital Annealer (NMcutDA) method recursively cuts a group of sequences into two and constructs a phylogenetic tree from the subclusters [1]. The normalized cut value (Ncut energy; defined in Shi and Malik 2000 [1] and explained in Eq. 4), a criterion for graph cut, was used for clustering criterion. Minimizing this criterion minimizes the similarity between subclusters and maximizes the similarity within subclusters at the same time when clustering sequences into two. In NMcutDA method, it first searches for minimum cut, which only minimizes similarity between subclusters, and then searches normalized cut during postprocessing (algorithms explained in materials and methods).

**Fig. 1:**
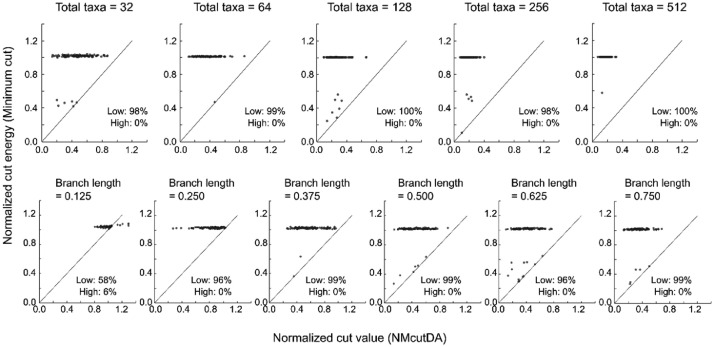
The Normalized cut energy of a similarity matrix derived from phylogenetic trees with different sizes of taxa or lengths of branches. A comparison was conducted between the normalized cut energy produced by the minimum cut and NMcutDA methods. The percentage of cases where the NMcutDA method showed less energy than the minimum cut is referred to as “Low,” and the percentage of cases where it showed more energy is referred to as “High.”

The Ncut energy was compared between the minimum-cut and postprocessed NMcutDA methods (Figure 1). Low/High, as shown in Figure 1, is the percentage of data for which the NMcutDA method had a lower/higher Ncut energy compared to that of the minimum cut. Top panel: Ncut energy produced from different numbers of taxa (32, 128, 256, and 512) with an average branch length of 0.500 [substitutions/site] (phylogenetic trees in the newick format are available in Supplementary Table 1). Bottom panel: Ncut energy produced from 32 sequences with different average branch lengths following a normal distribution (0.125, 0.250, 0.375, 0.500, 0.625, and 0.750 [substitutions/site]) (phylogenetic trees in the newick format are available in Supplementary Table 2 at https://data.mendeley.com/datasets/gjyhp3f8f3/1). Here, increasing the number of taxa increases the complexity of the minimum-cut problem and generally makes finding the minimum cut more difficult. Additionally, increasing the average branch length of the phylogenetic tree is equivalent to decreasing the similarity between sequences, thereby making construction of the phylogenetic tree more difficult [1].

**Fig. 2:**
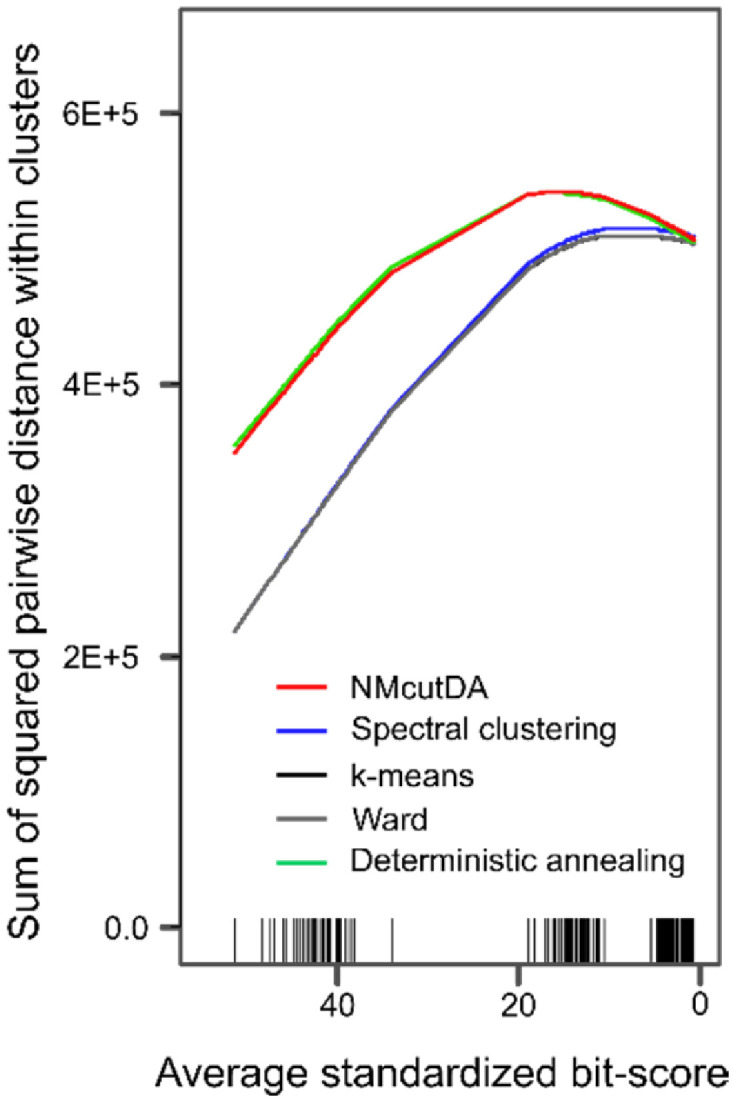
Comparison of different clustering methods based on k-means criteria (sum of squared pairwise distances within subclusters). The x-axis represents the average value of the standardized bit-score derived from each similarity matrix.

**Fig. 3:**
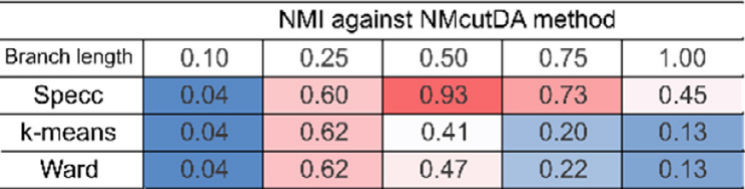
Comparison of normalized mutual information (NMI) scores between different clustering methods. The NMI score represents the similarity of two clustering results and was compared with the NMcutDA method. Higher scores are represented in red, while lower scores are represented in blue.

Next, the graph cuts of NMcutDA method were compared with existing clustering methods (spectral clustering, k-means, ward, and deterministic annealing) based on the Ncut energy. Specifically, phylogenetic trees were prepared with five different average branch lengths following a normal distribution, with a variance equal to half the average (0.10, 0.25, 0.50, 0.75, and 1.00 [substitution/site]). A comparison of the Ncut energy between the methods is shown in Figure 3A by Onodera et al. [1]. Next, the sum of the squared pairwise distances of the sequences within the same cluster was calculated for the similarity matrix, which is a criterion for k-means clustering (Figure 2). The x-axis represents the average standardized bit score, which is the average of the corresponding similarity matrix without diagnol elements (calculated as in Eq. 1). The ticks at the base of the graph represent the locations of the similarity matrices. In addition, we used normalized mutual information (NMI), which represents the similarity between two clustering results (details in Materials and Methods), to evaluate the similarity of cuts between the methods. The NMI between the spectral clustering/K-means/Ward and NMcutDA methods was calculated and is represented by a heat map (Figure 3).

**Fig. 4:**
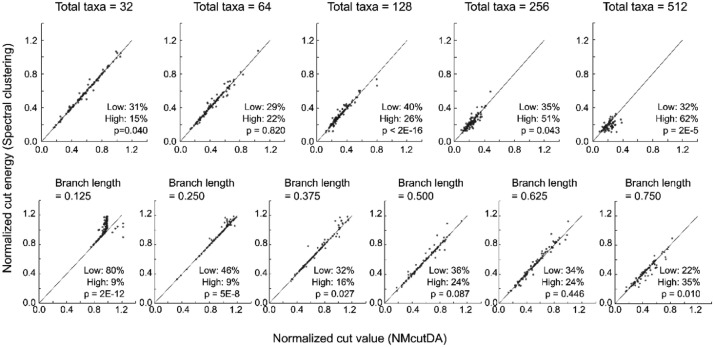
Normalized cut energy of a similarity matrix derived from phylogenetic trees with different sizes of taxa or lengths of branches. A comparison was conducted between the normalized cut energy produced by the spectral clustering and NMcutDA methods. The percentage of cases where the NMcutDA method showed less energy than the spectral clustering method is referred to as “Low,” and the percentage of cases where it showed more energy is referred to as “High.”

Both NMcutDA and spectral clustering methods minimize Ncut energy to determine the optimal graph cuts. Therefore, the Ncut energy was precisely compared between the NMcutDA and spectral clustering methods (Figure 4) [2]. Low/High is the percentage of data for which the NMcutDA method has a lower or higher energy than the spectral clustering method. P-values were calculated using the Wilcoxon signed-rank test. Top panel: Ncut energy produced from different taxa (32, 128, 256, and 512) with an average branch length of 0.500 (phylogenetic trees are available in Supplementary Table 3, https://data.mendeley.com/datasets/gjyhp3f8f3/1). Bottom panel: Ncut energy produced from 32 sequences with different average branch lengths (0.125, 0.250, 0.375, 0.500, 0.625, and 0.750) following the Yule model phylogenetic tree (phylogenetic trees are available in Supplementary Table 4, https://data.mendeley.com/datasets/gjyhp3f8f3/1). A Yule model with a birth rate of one was used to simulate a realistic phylogenetic tree.

**Fig. 5:**
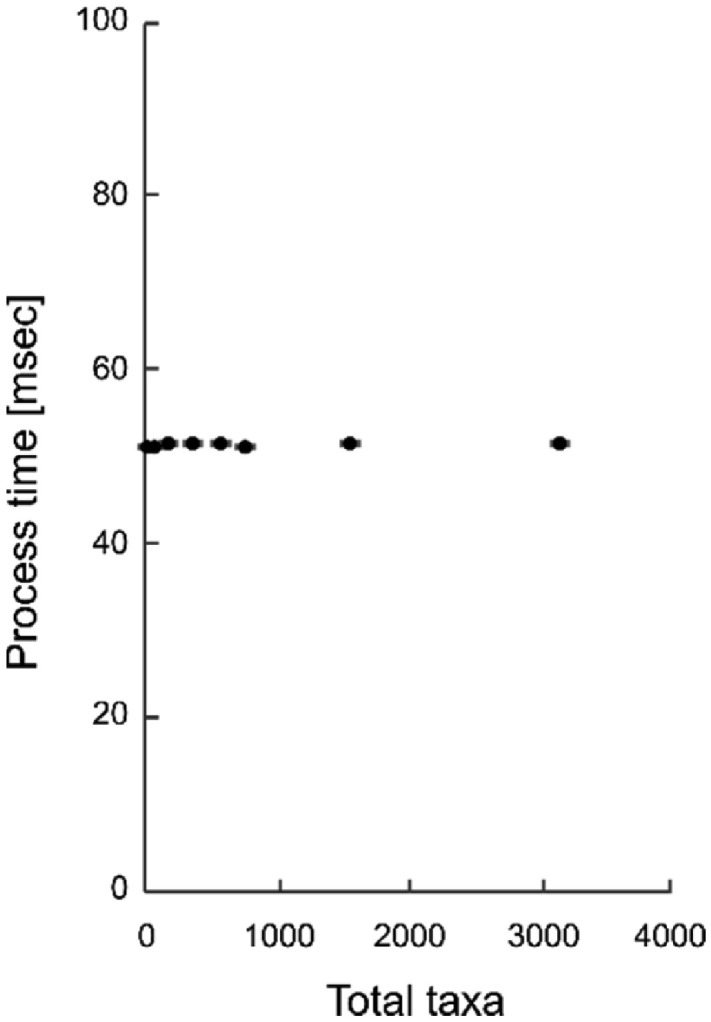
Processing time of the NMcutDA method for each cut with different numbers of total taxa.

The dependence of the NMcutDA processing time on the number of taxa is plotted in Figure 5. Error bars represent standard error of the mean (SEM) of 50 replicates.

**Fig. 6:**
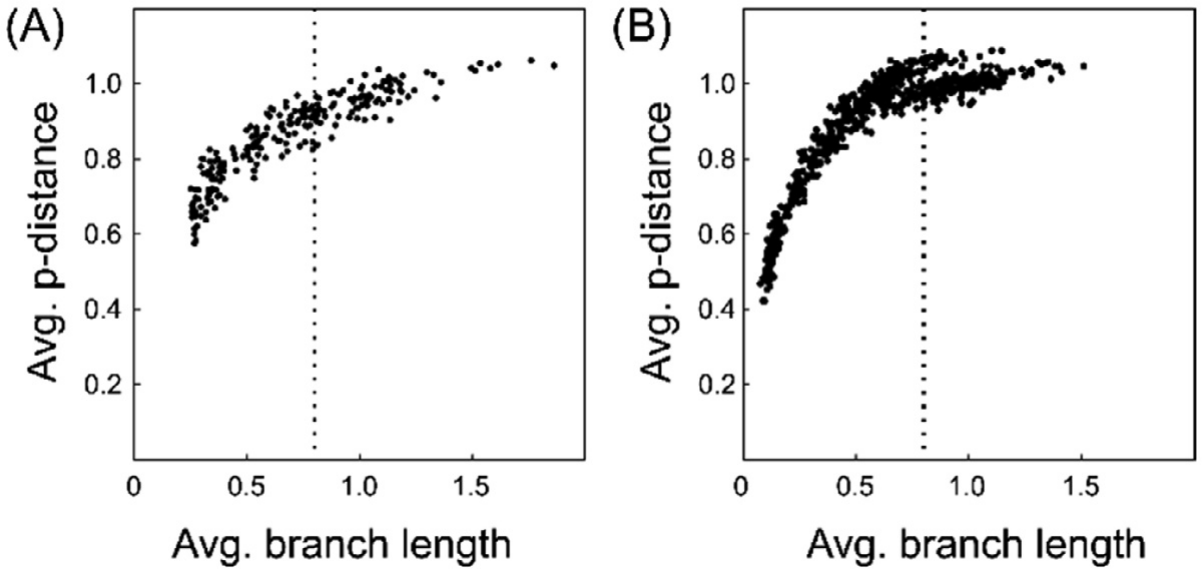
Average p-distance plotted against average branch length of the corresponding phylogenetic tree. The phylogenetic tree was either derived from (A) a yule model-based simulation or (B) the RAxML grove database.

**Fig. 7:**
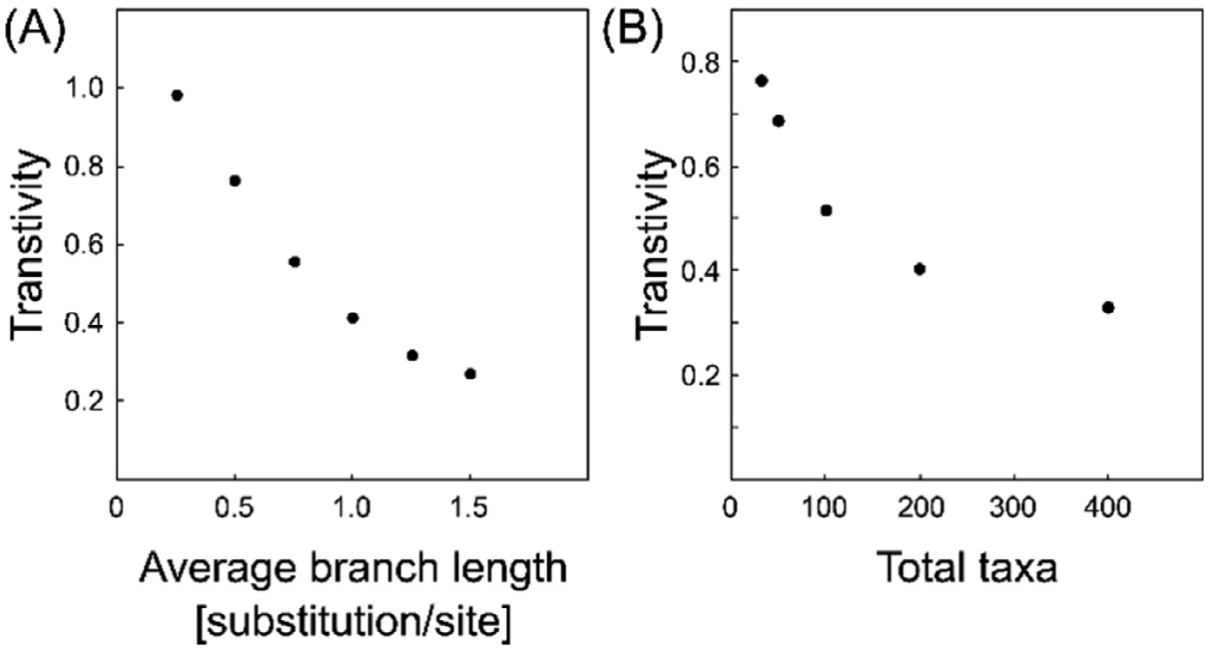
Transitivity plotted against (A) average branch length of the corresponding phylogenetic tree or (B) number of total taxa.

To understand the sequence similarity within each phylogenetic tree, the corresponding average p-distance to the average branch length of each Yule model and the RAxML-derived similarity matrix are shown in Figure 6 [3,4]. The p-distance is the simplest evolutionary distance and is the ratio of the difference between two sequences (a p-distance = 0 represents identical sequences). The left panel (A) shows the average p-distance of the phylogenetic trees generated under the Yule model (phylogenetic trees are available in Supplementary Table 4 at https://data.mendeley.com/datasets/gjyhp3f8f3/1). Right panel (B) shows the phylogenetic trees downloaded from the RAxML Grove database (phylogenetic trees are available in Supplementary Table 5 at https://data.mendeley.com/datasets/gjyhp3f8f3/1). Finally, the dependency of transitivity, which evaluates the interconnectivity of the graph (similarity matrix), on branch lengths and the number of sequences was calculated (Figure 7). The left panel (A) shows the relationship between transitivity and branch length. The right panel (B) shows the relationship between transitivity and number of sequences.

## 3 Experimental Design, Materials and Methods

### 3.1 Material and Methods

The NMcutDA method consists of three stages:1) reconstruction of the similarity matrix of the sequence set, 2) min-cut ($Min_{cut}$) of the similarity matrix using Fujitsu DA, and 3) standardization of the cut.

### 3.2 Reconstruction of the Similarity Matrix for a Set of Sequences

NMcutDA is a hierarchical clustering method based on the similarity matrix of sequences. First, the similarity between each sequence was calculated and a similarity matrix was generated. All-to-all pairwise alignment (PSA) was adopted in this study because it is more sensitive than multiple sequence alignment for calculating similarity between dissimilar sequences. Because PSA is not influenced by the presence of other sequences, it can be used when multiple sequence alignments fail to align sequences accurately [5,6]. The PSA results were used to calculate bit scores. The bit score can be calculated based on the amino acid similarity matrix (BLOSUM62), with a higher similarity between sequences represented by a larger value [7,8]. The bit score was then normalized as described by Dufour *et al*. (2010) [9]. The normalization of the bit score between sequences i and j is defined as

(1) $$Norm_{bit\left(i,j\right)}=\frac{bit\left(i,j\right)}{mean\left\{bit\left(i,i\right),\;bit\left(j,j\right)\right\}}*100,$$

where bit(i,j) represents the bit score between sequences i and j. Unlike Dufour *et al.* [9], the bit score was normalized to values ranging from 0 to 100, with identical sequences having a similarity of 100. This normalization was conducted because the DA permits only integer values. Under default settings, bit(i,j) was calculated using blastp in BLAST + 2.12.0, with an e-value of 10 as the lower limit. When two sequences did not reach an e-value of 10, $Norm_{bit(i,j)}$ was set to zero. The similarity matrix used in this study was produced using the following steps.

1. Sequences were generated or acquired in fasta format (steps for the generation of sequences used in this study are described below).
2. PSA was conducted and bit scores were calculated within the sequences using BLAST +, with the e-value cut-off option set to 10. BLOSUM62 was used as an amino acid similarity matrix.
3. For all sequence pairs i,j, $Norm_{bit(i,j)}$ was calculated and formatted as a matrix. This matrix was used as the input for DA.

### 3.3 Minimum Cut ($Min_{cut}$) of Similarity Matrix E Through Fujitsu Digital Annealer

The goal is to partition the sequence set into two subgroups based on the similarity matrix, where the elements are $Norm_{bit(i,j)}$. In particular, the similarity matrix can be denoted as a graph structure G=(V,E), where V denotes the nodes (or sequences) and E is the set of edges (or the similarity matrix). A phylogenetic tree was then reconstructed by recursively performing cuts on the resulting subgroups.

To search for a nearly optimal cut, we adopted a second-generation DA, a quantum-inspired digital circuit optimized for combinatorial optimization problems, using the Markov chain Monte Carlo method [10]. Formulating the combinatorial optimization problem in a formula that allows the DA to compute it enables the application of a high-speed DA algorithm. The DA employs hardware-based techniques, such as parallel search, escape from a local solution, and replica exchange, to accelerate the solution to optimization problems. It is capable of handling problems up to an 8,192-bit scale with fully coupled connectivity and high coupling resolution and can be applied to various real-world combinatorial optimization problems, such as drug discovery and delivery planning [10]. With the advent of third- and fourth-generation DA, 100,000 bits can be handled.

In the DA, the objective function for the cut must be expressed in binary quadratic form (or Quadratic Unconstrained Binary Optimization; QUBO). Therefore, the following objective function $Min_{cut}$ is formulated for a set of n sequences.

(2) $$Min_{cut}=\sum_{i=1}^{n-1}\sum_{j=i+1}^{n}d_{ij}{\left(x_{i}-x_{j}\right)}^{2},\;x_{i}=\left\{0,1\right\}$$

In the above function, $x_{i}$ is a binary variable for sequence i, which takes the value of 0 or 1. This binary value serves as a label for both subgroups. $d_{ij}$ is the similarity between sequences i and j retrieved from the similarity matrix, with the normalized bit scores as elements. In this equation, the similarity $d_{ij}$ is added to $Min_{cut}$ only when $x_{i}$ and $x_{j}$ are 0 and 1, or vice versa (i.e., when they are assigned to different subgroups). Among the combinations of $x_{i}$ and $x_{j}$, the DA searches for the minimum value of $Min_{cut}$, as such, a value makes the two subgroups the most dissimilar. Notably, DA generally performs comparably to state-of-the-art dedicated solvers when solving the MinCut problem in terms of speed and solution quality [10].

The minimum value of $Min_{cut}$ was equal to the minimum cut in the graph-cut problem. The $Min_{cut}$ was used in our previous study and successfully partitioned a set of sequences equivalent to those obtained using the neighbor-joining method and principal component analysis [11]. However, cutting was conducted only once because $Min_{cut}$ has the critical problem of making biased cuts unsuitable for phylogenetic tree reconstruction.

Generally, the minimum cut suffers from an unnatural cut that partitions out small sets of nodes because the total weight of the edges can easily take a small value in the case of a small number of edges to be cut [2]. This trait interferes with the reconstruction of the phylogenetic tree by removing a small set of nodes from the sequences. We have shown that $Min_{cut}$ consistently cuts only a single node, despite generating phylogenetic trees under various conditions (shown in Fig 2A, B of Onodera et al. [1]). Therefore, a constraint term was added to the second term of $Min_{cut}$ to control the number of nodes to be partitioned.

(3) $$Min_{cut}=\sum_{i=1}^{n-1}\sum_{j=i+1}^{n}d_{ij}{\left(x_{i}-x_{j}\right)}^{2}+\alpha {\left(\sum_{i=1}^{n}x_{i}^{1}-c\right)}^{2}$$

The constraint term at the second term consists of a positive integer constant α, a total number of nodes labelled “1” $\sum_{i=1}^{n-1}x_{i}^{1}$, and an arbitrary natural number c. Thus, the second term has a value greater than or equal to zero. Therefore, if this constraint term takes a value greater than zero, $Min_{cut}$ becomes large, which is disadvantageous for determining the minimum cut value. The constraint term can only take the minimum value of zero if c and $\sum_{i=1}^{n-1}x_{i}^{1}$ are equal. For example, if c is set to 5 and $\sum_{i=1}^{n-1}x_{i}^{1}=3$, the second term now has an excess energy of 2α (which can be cancelled out if $\sum_{i=1}^{n-1}x_{i}^{1}=5$). Therefore, by determining c, the total number of nodes with the “1” label can be indirectly controlled. α was determined by monitoring the energy of the second term, and if not 0, the $Min_{cut}$ was restarted with α+500. For the third and fourth generations of the DA, α can be automatically determined. The DA preprocessed the input similarity matrix as follows and performed annealing. These steps are automated, and users only need to provide similarity matrix.

1. For each sequence i of the similarity matrix, binary label $x_{i}\in \left\{0,\;1\right\}$ was produced.
2. Eq. 3 was expanded together with the similarity matrix, where each element was $d_{ij}$. α=100,c=1 were set as the initial values. The expanded result was in binary quadratic form, which can be handled by the DA. This step was performed using the PyQUBO library and DA.
3. Annealing was conducted using the DA under default parameters. Then, the minimum $Min_{cut}$ value and value of the second term in Eq. 3 (penalty energy) were returned.
4. If the penalty energy was a non-zero value, we returned to step 2 and added 500 to value α. A non-zero value indicates that the cut did not occur for a predefined number of nodes in each subclusters.
5. If the penalty energy was zero, the minimum $Min_{cut}$ value was pooled.
6. One was added to constant c until c=n/2 or c=(n+1)/2, after which we returned to step 2.
7. For the minimum $Min_{cut}$ value of each c, the standardization step described in the following section was applied.

### 3.4 Standardization of $Min_{cut}$

In this study, we used a criterion called $N_{cut}$ to partition the similarity matrix into two disjoint sub-clusters. $N_{cut}$ measures not only the total dissimilarity between two subclusters but also the total similarity within each subcluster and frequently provides a natural cut compared to $Min_{cut}$ [2]. $N_{cut}$ can be directly calculated using $Min_{cut}$. Assuming that a graph with nodes (or sequences) V is partitioned into A and B subgroups (A∪B=V), the following equation is defined:

(4) $$N_{cut}=\frac{Min_{cut}}{assoc\left(A,\;V\right)}+\frac{Min_{cut}}{assoc\left(B,V\right)}$$

$assoc\left(A,V\right)=\sum_{u\in A,t\in V}d_{ut}$ is the total similarity between each node in subgroups A and V. assoc(B,V) can be defined similarly. $N_{cut}$ is a criterion that chooses a cut that increases the similarity within a group (assoc(A,V) and assoc(B,V)) while keeping the similarity between subgroups low ($Min_{cut}$). Note that when utilizing $N_{cut}$ as a criterion, the objective function for DA must be expressed in a binary quadratic form; thus, $N_{cut}$ cannot be directly obtained, but only by post-processing $Min_{cut}$.

In summary, 1) $Min_{cut}$ is first searched for all possible cut patterns in DA, and this can be accomplished by changing c in Eq. 3, which controls the number of nodes to be partitioned. 2) Then, $N_{cut}$ was then calculated using Eq. 4: 3) Among the derived $N_{cut}$ energies, the one with the minimum $N_{cut}$ energy was selected as the nearly optimal cut. Twelve parallel runs were conducted simultaneously to verify convergence of the search for the optimal cut. These steps were applied recursively to the derived subclusters until a dendrogram (phylogenetic tree) was obtained. We call our proposed method the normalized minimum cut by Digital Annealer (NMcutDA) method. The below shows the listed algorithms from choosing optimum cut for NMcutDA method to constructing phylogenetic trees. Note that these steps are also automated.

1. For the minimum $Min_{cut}$ value of each c, $N_{cut}$ was calculated using Eq. 4.
2. The sequences were divided into two sub-clusters with the smallest $N_{cut}$ value, representing a nearly optimal clustering result.
3. The minimum $Min_{cut}$ value for each sub-cluster was searched until n=1or2 in all clusters.
4. Phylogenetic trees in newick format were created by reconstructing the branches using the clustering result.

### 3.5 Generation of Simulated Phylogenetic Trees and Sequences

A phylogenetic tree was randomly generated to verify the accuracy of the phylogenetic tree reconstruction under controlled parameters. The sequences based on the true phylogenetic tree were simulated and verified to reconstruct the original phylogenetic tree. For further analysis of the accuracy of the reconstructed phylogenetic tree, see Onodera et al. [1]. First, the *rtree* function under the default parameters of the ape package v5.6-1 or the *sim.bd.taxa* function (lambda = 1, mu =1, frac=1, complete=FALSE, stochsampling=FALSE) of the TreeSim package v2.4 was used to generate a phylogenetic tree [3,11]. The *r-tree* function can be used to reconstruct a phylogenetic tree with a random shape. The lengths of the branches followed a normal distribution with specified means (set of [0.125, 0.250, 0.375, 0.500, 0.625, and 0.750] or [0.10, 0.25, 0.50, 0.75, and 1.00]) and half the variance. Fifty phylogenetic trees were constructed for the *rtree* function (Supplementary Table 1). The *sim.bd.taxa* function produced a phylogenetic tree using the Yule process (birth rate = 1). Branch lengths with specified means (0.125, 0.250, 0.375, 0.500, 0.625, and 0.750) follow an exponential distribution (λ = 2) [12]. One hundred phylogenetic trees were replicated for the *sim.bd.taxon* function (Supplementary Table 2; https://data.mendeley.com/datasets/gjyhp3f8f3/1). Based on the phylogenetic trees, we simulated sequence data consisting of 500 residues using INDELible v1.03 [13]. The amino acid substitution model used was as follows: WAG+Γ (k = 5 categories) + I (1% invariant sites). The power-law insertion/deletion length distribution was set to a default value of a = 1.7. The average numbers of insertions and deletions were set to 2% of the average number of substitutions.

### 3.6 RAxML Grove Database Usage

The RAxML Grove database is a data server containing more than 60,000 phylogenetic trees constructed from real data [4]. These phylogenetic trees were obtained using the maximum likelihood method from MSAs submitted by users on the RAxML/RAxML-NG online server. Taxon names were anonymized, and MSAs could not be obtained; only the topologies of the phylogenetic trees were available. These phylogenetic trees are useful when benchmarking the development of construction methods because the parameters do not need to be considered when simulating the trees. Therefore, we considered it a suitable database to use for sampling the topology of phylogenetic trees based on real sequences. In this study, we generated sequences using INDELible based on trees from the RAxML Grove database and tested the ability of the NMcutDA method. We filtered the database (NUM_TAXA:20–40, BRANCH_LENGTH_MEAN:0.25–0.75, BRANCH_LENGTH_VARIANCE < 0.25) and obtained 78 phylogenetic trees. Their branch lengths were then doubled (branch length = 0.50–1.50) and tripled ((branch length = 0.75–2.25)) and 224 phylogenetic trees were used for analysis (Supplementary Table 3 of https://data.mendeley.com/datasets/gjyhp3f8f3/1).

### 3.7 Evaluation on a Graph Structure

The NMI was used to evaluate the similarity of the cuts between the methods. We applied *the normalized_mutual_info_score* function (average_method='arithmetic') of scikit-learn v0.22.1 to the two cut results as the input in Python [14]. Transitivity measures a graph's interconnectivity by calculating the ratio of the observed number of closed triads (triangles) to the maximum number of closed triads. This was calculated from the similarity matrix by using the *transitivity_undirected* function (under default parameters) in iGraph v1.2.11 in R [15].

### 3.8 Clustering by k-means, Ward, Spectral Clustering, and Deterministic Annealing Method

For k-means clustering and the ward method, the *k-means* (centers = 2, nstart = 100) and *hclust* functions (under default parameters) of the stats package were used. For the spectral clustering method, *spectral clustering* (n_clusters = 2) of scikit-learn v0.22.1 was used. The similarity matrix calculated using Eq. 1 was used as the affinity matrix for *SpectralClustering. Deterministic annealing* (n_clusters = 2, distribution was set to the same as that of the NMcutDA method) in the size-constrained clustering package (v0.1.1) was used for the deterministic annealing method.

### 3.9 Computer Environment

To evaluate the NMcutDA method, we used CPLEX and other existing methods (including K-means, Ward, spectral clustering, and deterministic clustering methods). CPLEX is a general-purpose exact solver based on linear programming with a guaranteed optimum solution (Cplex, 2009). V12. 1: User manual for CPLEX; 46. This Corporation, 157) It was calculated using CentOS 7.6.1810, Intel Xeon CPU E5-1650, 3.60 GHz (six cores, 12 threads), Python 3.7.10, and CPLEX 12.9.0.0. All other calculations were performed using Windows 10 12th Gen Intel(R) Core(TM) i9-12900K 3.19 GHz, except for the DA and Python operations. The DA has already been applied to problems in the real world such as drug discovery and delivery planning. The user guide and API references for DA users are available online. The Digital Annealer User guide is available at https://portal.aispf.global.fujitsu.com/apidoc/da/jp/da-guide-en.html, and the Digital Annealer API Reference is available at https://portal.aispf.global.fujitsu.com/apidoc/da/jp/api-ref/da-qubo-en.html. The Fujitsu Digital Annealer uses quantum-inspired computing technology and is available at https://www.fujitsu.com/global/services/business-services/digital-annealer/.

## CRediT authorship contribution statement

**Wataru Onodera:** Conceptualization, Methodology, Investigation, Writing – original draft. **Nobuyuki Hara:** Software, Investigation, Validation. **Shiho Aoki:** Software. **Toru Asahi:** Resources. **Naoya Sawamura:** Conceptualization, Resources, Writing – review & editing, Funding acquisition, Supervision.

## Declaration of Competing Interest

The authors declare that they have no competing financial interests or personal relationships that may have influenced the work reported in this study.

## Data Availability

Data for: Phylogenetic tree reconstruction via graph cut presented using a quantum-inspired computer (Original data) (Mendeley Data)

## Appendix Supplementary materials

Image, application 1

Image, application 2

Image, application 3

Image, application 4

Image, application 5
